# Ethnobotanical plants used in the management of symptoms of tuberculosis in rural Uganda

**DOI:** 10.1186/s41182-021-00384-2

**Published:** 2021-11-22

**Authors:** Christine Oryema, Karlmax Rutaro, Sam William Oyet, Geoffrey Maxwell Malinga

**Affiliations:** 1 Department of Biology, Faculty of Science, Gulu University, P.O. Box 166, Gulu, Uganda; 2 Department of Biochemistry and Sports Science, College of Natural Sciences, Makerere University, P. O. Box 7062, Kampala, Uganda

**Keywords:** TB, Treatment, Traditional Medicine, Knowledge, Practitioners, Medicinal plants, Uganda

## Abstract

**Background:**

Tuberculosis (TB) caused by *Mycobacterium tuberculosis* is the 13th leading cause of death worldwide. The emergence of multidrug-resistant TB (MDR-TB) poses a major health security threat. Plants have traditionally been used as a source of medicine, since olden days and 80% of the communities in Africa still rely on herbal medicines for their healthcare. In many parts of Uganda, some plants have shown ethno-pharmacological prospects for the treatment of TB, and yet they have not been fully researched.

**Aim:**

This study aimed to document plant species used traditionally by the herbalists and non-herbalist communities of Kitgum and Pader districts for managing symptoms of TB.

**Methods:**

An ethnobotanical study was carried out in 42 randomly selected villages in Kitgum and Pader districts between August 2020 and January 2021. Information was obtained by administering semi-structured questionnaires to 176 respondents identified by snowball and random sampling methods. Data were analysed and presented using descriptive statistics and Informant Consensus Factor (ICF).

**Results:**

Overall, only 27% of the respondents were knowledgeable about plants used for managing symptoms of TB. Nine plant species belonging to six families (Mimosaceae, Apiaceae, Lamiaceae, Rutaceae, Loganiaceae and Rubiaceae) were used to manage symptoms of TB. The most representative family was Rutaceae with three species, followed by Rubiaceae (two species) and the rest of the families were represented by one species each. The most frequently recorded species were *Steganotaenia araliacea* Hochst. (8.5%), *Gardenia ternifolia* Schumach. & Thonn (6.8%) and *Albizia adianthifolia* (Schum.) W.Wight (6.8%). Most of the medicinal plants were trees, and roots (69%) were the most frequently plant part used, followed by the bark (16%) and leaves (15%). The most common method of preparation was by pounding and mixing concoction with water. The administration of the concoctions was mostly done orally.

**Conclusions:**

The results established the existence of few medicinal plants for managing symptoms of TB among the Acholi communities which could be used in developing new, effective plant-based antimycobacterial drugs. The few plants mentioned might face conservation threats due to exploitations of the roots. Phytochemical and toxicological studies are recommended to identify active compounds responsible for antimycobacterial activity.

## Background

Tuberculosis (TB) is an ancient communicable disease that has been in existence over the past millennia and remains a major global public health burden, with one-third of the world’s population currently infected and more than 1.3 million deaths per year [1, 2]. It is a contagious disease caused by *Mycobacterium tuberculosis*. It usually attacks the lungs, although it can also spread to other parts of the body, and is transmitted through coughing and sneezing [2]. In most Asian and some African countries, the prevalence of TB per 100,000 population peaks among those aged 35‒54 years [1]. This covers the productive age group, which consequently affects the growth of any country. Globally, an estimated 10 million people developed active TB disease in 2019, with 1.4 million TB deaths [1]. Progress in achieving the United Nations (UN) General Assembly End TB targets remains slow.

Uganda is among the 30 high TB/HIV burden countries accounting for 90% of the global TB burden [2] with an estimated TB prevalence and incidence at 253 cases per 100,000 population and 234 cases per 100,000 annually, respectively [3, 4]. Countrywide, about 7% of the health centres are reportedly providing TB treatment services but lack diagnostic services [5]. This could mean that many people within the communities go undiagnosed and continue to transmit TB among themselves. Northern Uganda with a population of about 6–7 million has a high TB prevalence up to about 8% [6]. Currently, treatment for TB is administered orally and comprises a combination of some or as single therapies of rifampicin, isoniazid, ethambutol and pyrazinamide which are taken for 6 months [7]. Even though this treatment is effective, the utilization of this regimen is challenged by either low or lack of adherence to prescribed therapies, and inefficient healthcare structures [4] which have contributed to the rise of multi-drug resistant strains (MDR) of *M. tuberculosis* [8, 9]. The simplicity with which TB infection spreads, for instance, by inhalation of droplets nuclei 2–5 mm in diameter containing as few as 1–3 bacilli has helped to sustain this scourge at current levels [8]. However, although millions of people are successfully getting treatment for TB each year, there are still large gaps between the number of people diagnosed and reported, and those accessing treatment [4]. The declining access to adequate TB drugs in the rural health centres calls for an extensive search for complementary, easily accessible and affordable TB drugs which could shorten treatment duration [10]. The use of plant-based drugs could be a complementary drug to treat both resistant and susceptible forms of TB. Medicinal plants have been in use for the treatment of humans and animals in many tribal cultures, and traditional knowledge related to this has been passed on from one generation to the next [11, 12]. It is estimated that nearly 80% of the world’s total population particularly in developing countries depend on traditional medicinal plants and products for their primary healthcare needs [12, 13], due to the high cost of western-style medicines. Uganda’s enormous wealth of plant resources constitute an important part of health care especially for the rural poor [14]. Several ethnobotanical studies have documented medicinal plants being used to treat several diseases [12, 14–17].

For 20 years (1986–2006), the Acholi sub-region in Northern Uganda experienced conflict as a result of fighting between the Uganda Peoples Defense Forces (UPDF) and the Lord’s Resistance Army (LRA) [18]. Among the many impacts of the war, this also devasted all social services including health systems. The displacement and overcrowding in the IDP camp, coupled with poor sanitation, poor medication and reckless social life escalated health conditions including TB and HIV/AIDS infections. When the community returned to their original homestead in 2006, they continued mixing freely with no precautionary measures hence increasing chances of infection. Inadequate functional medical facilities and poor access to healthcare in rural communities cause many people to seek herbalists who use medicinal plants as remedies for various disease conditions [19, 20]. Due to the prolonged war in the Acholi sub-region, limited research on ethnobotanical plant resources has been undertaken. Most previous studies have focused mostly on humanitarian and political aspects [21, 22] and the use and consumption of wild edible plants [23–25]. However, in many parts of Uganda, some plants have shown ethno-pharmacological prospects for the treatment of TB, and yet they have not been fully researched. Unfortunately, the knowledge of which medicinal plants are used in treatments of TB is not well understood. Consequently, there is a need to assess the biodiversity-based cultural knowledge in the Acholi sub-region to ascertain the available medicinal plants that could be exploited in developing new drugs for managing TB and its associated symptoms. This study aimed at documenting plant species used traditionally by herbalists and non-herbalists in Kitgum and Pader districts for managing symptoms of TB, sources of plant knowledge, parts used, habits, side-effects, methods of preparation and modes of administration.

## Methods

### Description of the study area

**Fig. 1:**
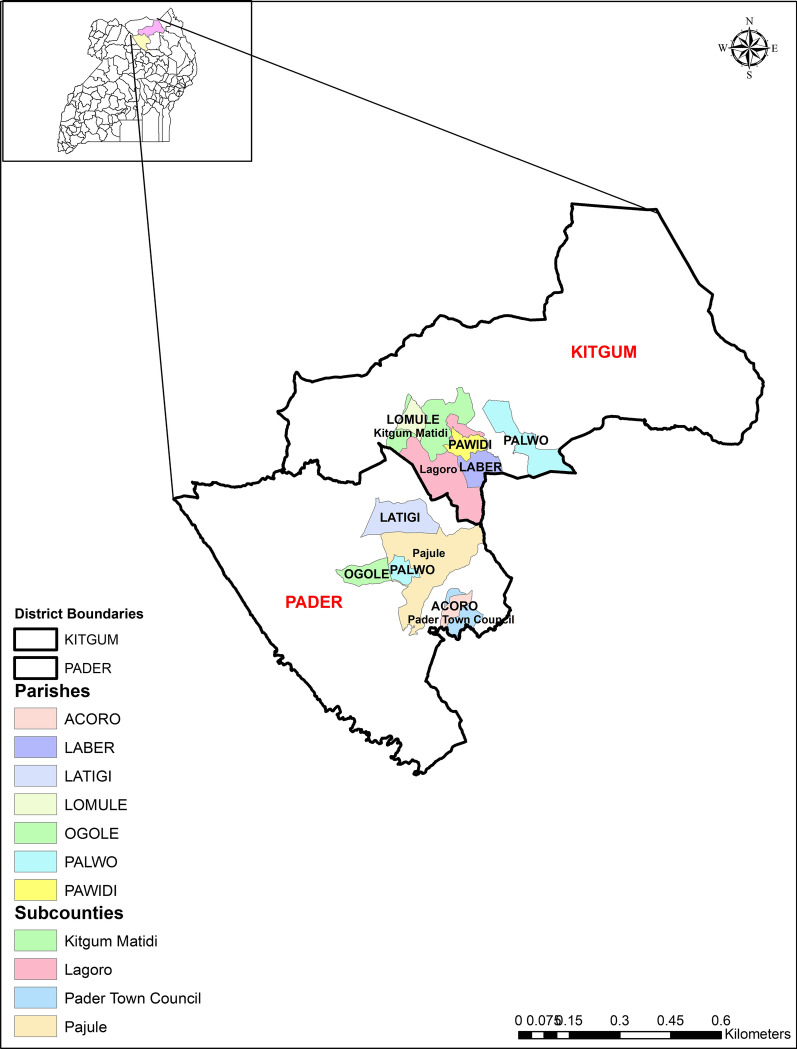
Location of parishes surveyed for plants used in managing symptoms of TB in Kitgum and Pader districts, Uganda. The map was created by the authors using ArcGIS version 10.3.1

This ethnobotanical survey of plants used in the management of TB symptoms was undertaken in Kitgum and Pader districts in the Acholi sub-region of Uganda (Fig. 1). In general, the Acholi sub-region is categorized as a tropical dry climate similar to the southern part of Sudan. The land is generally well watered in the rainy season and fertile. Most of the land is covered by woody and green vegetation. The total population in Kitgum is 204,048 and that of Pader is 178,004 persons [26]. We selected Kitgum and Pader districts, because these areas are situated at the border and periodically experience an influx of refugees escaping conflicts in Southern Sudan. Moreover, these are unscreened for several diseases hence exposing the population to increasing risks of contraction of diseases including TB. High levels of poverty, poor access to modern health facilities also characterize the selected locations, causing many people to seek herbal remedies for the treatment of various disease conditions.

### Population and sample size

The expected sample size of 384 respondents was calculated using the Krejcie and Morgan Table [27]. The respondents included herbalists and non-herbalists aged 15 years and above, covering both females and males of different age groups. However, due to the constraints of time and resources, we interviewed only 176 respondents including 20 herbalists and 156 non-herbalists (i.e., local people who regularly use plants for medicinal purposes) from both Kitgum and Pader districts.

### Data collection and selection of study sites and participants

The field survey for this study was conducted from August 2020 to January 2021. From each district, two sub-counties were selected, from which four parishes were randomly selected, and eventually, five to six villages were selected from each parish, resulting in a total of 42 villages. In Kitgum district, Kitgum Matidi and Lagoro sub-counties were included covering Lumule and Paibony, Lukwor and Lukwir parishes, respectively. While in Pader district, Pader Town Council (within the parishes of Acoro and Luna) and Pajule Town Council (within the parishes of Ogole and Olwo) were included. Purposive and snowball sampling method was used in the identification of the herbalist respondents. The first respondent was selected by the village/community leaders from each parish and village based on their reputability and ability to portray good traditional herbal medicine knowledge, and the subsequent respondents were identified by fellow herbalists. The non-herbalists were selected by a simple random sampling technique [28] from the village household register. We selected both herbalists and non-herbalists to ensure that our sample included representatives of the whole community. For each respondent, we recorded information on the socio-demographics including location, gender, age, marital status, age, level of education and occupation and how they acquired knowledge about medicinal plants. Before the interview, the purpose of the study was explained to the respondents and voluntary written prior informed consent and assent were obtained. The respondents were assured of the confidentially of the information and that the data would be used strictly for academic purposes. Ethnobotanical data were collected using semi-structured questionnaires in Acholi, the local language of the respondents and was facilitated by local field assistants who are fluent in both Acholi and English. The questionnaire included information on the local names of the medicinal plants used in TB treatment, the plant parts used, life form, the method of preparation and mode of administration of the herbal remedy. Voucher specimens of all plant species mentioned were collected, pressed and identified by a qualified taxonomist by comparing with herbarium specimens at the Makerere University herbarium (MUH). The catalogue of life (https://www.catalogueoflife.org/), the Flora of Tropical East Africa and the Angiosperm Phylogeny Group APGIV [29] were used to confirm the botanical names, families and authorities.

### Statistical analyses

The data were entered in Microsoft Excel Program, coded, and exported to SPSS software for analysis. Descriptive statistics such as frequencies and percentages in SPSS for Windows, version 26 was used to summarize ethnobotanical and respondents’ socio-demographic data. The Informant Consensus Factor, ICF [30, 31] was computed to find out the homogeneity in the ethnomedicinal information from the respondents. ICF was computed using the formula; ICF = Nur − Nt/(Nur − 1), where “Nur” refers to the total number of use reports for each disease cluster and “Nt” refers the total number of species in each use category. The ICF values range from 0 to 1; high ICF values are obtained when only one or a few plant species are reported to be used by a high proportion of informants to treat a particular category, whereas low ICF values indicate that informants disagree over which plant to use.

## Results

### Socio-demographic characteristics of the respondents

Out of a total of 176 respondents interviewed, the majority (65%) were females (Table 1). The majority of respondents were in the age bracket 36–45 years (34%, average age 40 years) and were mostly married (61%). The least was in the bracket 15–20 years (2.3%). Most (66%) respondents had attained primary level education and the majority were peasant farmers (82%) (Table 1).

**Table 1:** Socio-demographic characteristics of the respondents (*n* = 176)

Characteristic	Frequency	Percentage
Gender		
Male	61	35
Female	115	65
Age (years)		
15–20	4	2.3
21–25	48	27
26–35	28	16
36–45	59	34
Education		
None	45	26
Primary	116	66
Secondary/institution	15	8.5
Religion		
Catholic	84	48
Anglican	76	43
Muslim	12	7
Pentecostal	4	2
Occupation		
Peasant farmer	144	82
Pupils/students	32	18
Marital status		
Single	44	25
Married	108	61
Divorced/separated	24	14

### Plant species used by the healers and resource users to treat tuberculosis

**Fig. 2:**
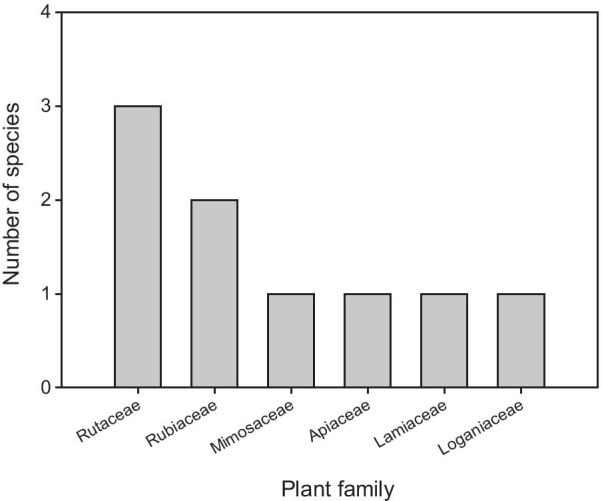
Number of plant species per family used for managing TB symptoms in Kitgum and Pader districts, Uganda

A total of nine plant species belonging to six families (Mimosaceae, Apiaceae, Lamiaceae, Rutaceae, Loganiaceae and Rubiaceae) and nine genera were reported to manage symptoms of TB in both study areas (Table 2). The most representative family was Rutaceae with three species, followed by Rubiaceae (two species) and the rest of the families were represented by one species each (Fig. 2). The most frequently recorded species were *Steganotaenia araliacea* Hochst. (8.5%), *Gardenia ternifolia* Schumach. & Thonn (6.8%) and *Albizia adianthifolia* (Schum.) W.Wight (6.8%) (Table 2).

**Table 2:** Plant species and associated information reported by respondents as used for treating Tuberculosis in the Acholi sub region, northern Uganda

Plant Family	Plant name	Local name (Acholi)	Voucher no	Habit	Parts used	Frequency of mention	Modes of preparation	Modes of Administration, quantity and	Side effects	Reference websites
Mimosaceae	*Albizia adianthifolia* (Schum.) W.Wight	Ayekyek	ORC02	Tree	R, B	12	Obtain the roots or the bark of this plant, mix it with the roots of Olwiro, pound together, mix with water to obtain a concoction and sieve	Drink one small cup (1–5mls) of the mixture/concoction 3 times a day for a month	none	http://bitly.ws/fNiD
Apiaceae	*Steganotaenia araliacea* Hochst	Olwiro	ORC01	Shrub	R	15	Pound the roots of this plant together with the roots or bark of Ayekyek (*Albizia adianthifolia*) and mix with water, sieve to obtain the concoction	Drink one small cup (6–10mls) of the mixture/concoction 3 times a day for a month	none	http://bitly.ws/fNgM
Lamiaceae	*Ocimum gratissimum* L	Yat aona opio	ORC08	Herb	R	1	Pound the roots, mix with water to obtain a concoction	Drink > 10mls (a cupful) of the concoction daily for 1–2 months	none	http://bitly.ws/fNhA
Rutaceae	*Zanthoxylum leprieurii* Guill. & Perr	Kicuk	ORC04	Tree	R, B	4	Pound roots and bark mix with warm water	Drink a 6–10mls (2 table spoonful) three times a day for 2 weeks	none	https://rb.gy/av5z7f
	*Zanthoxylum chalybeum* Engl	Roki	ORC09	Tree	R	1	Pound the roots mix with water and drink	Drink 10mls of the concoction three times a day until cure	Causes dizziness if drunk with cold water	http://bitly.ws/fNh2
	*Hallea stipulosa* (DC.) J.-F.Leroy	Oculup	ORC07	Tree	R	1	Pound the roots of Oculup together with lum layib oyoo, sieve and drink daily	Drink 6–10 mls concoction three times a day for 2 weeks		http://bitly.ws/fNhK
Rubiaceae	*Gardenia ternifolia* Schumach. & Thonn	Odwong	ORC03	Tree	R	12	Obtain three sets of roots of the same plant species but from different locations. Mix these roots in same proportions and pound finely and mix with cold water or warm look water	Drink one cupful (> 10mls) of the concoction twice a day for 1–2 weeks	none	http://bitly.ws/fNhr
	*Sarcocephalus latifolius* (Sm.) E.A.Bruce	Omwunyu	ORC05	Tree	R, B,L	1	Pound roots and bark, mix with 250mls of warm water to obtain a concoction	Drink 1/3 of a small cup 2 times daily for 1 month (but there is no specific dose locally)	none	http://bitly.ws/fNio
Lagniaceae	*Strychnos innocua* Delile	Alingkwalo	ORC6	Tree	R, B,L	1	Pound roots and bark mix with 250mls of warm water	Drink 1.5mls of the concoction 2 times daily for 1 month	none	http://bitly.ws/fNif

*ORC*  Oryema Christine, *R* root bark, *B* Stem bark, *L* leaves

**Fig. 3:**
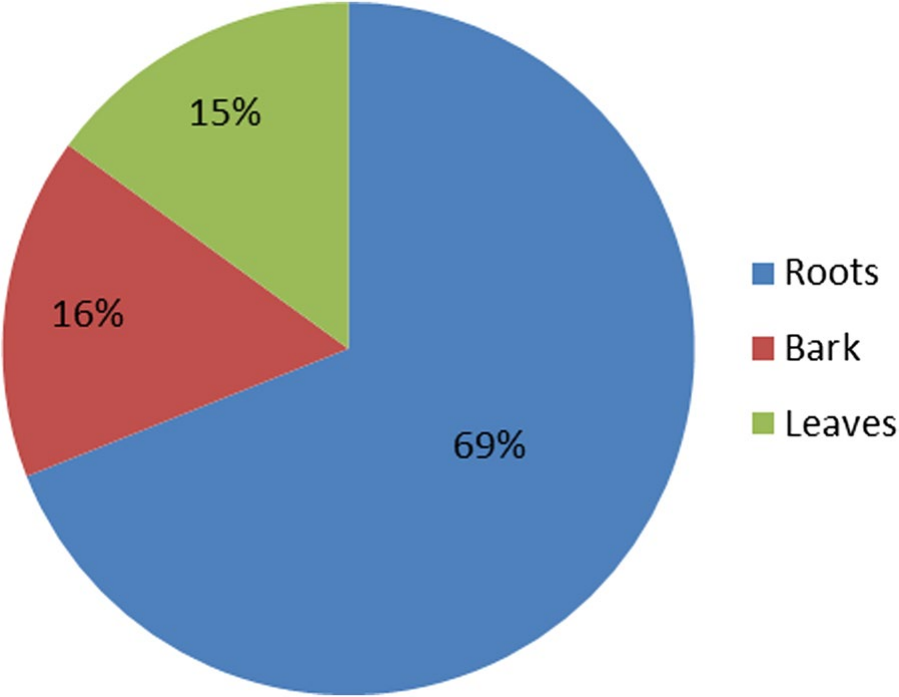
Percentage of plant parts used by the herbalist and non-herbalist for TB remedy in Kitgum and Pader districts, Uganda

The ICF value for management of TB was 0.83. Most of the medicinal plants were trees (seven species) and root was the most (69%) frequently plant part used for management of TB, followed by the bark (16%) and leaves (15%, Fig. 3).

**Fig. 4:**
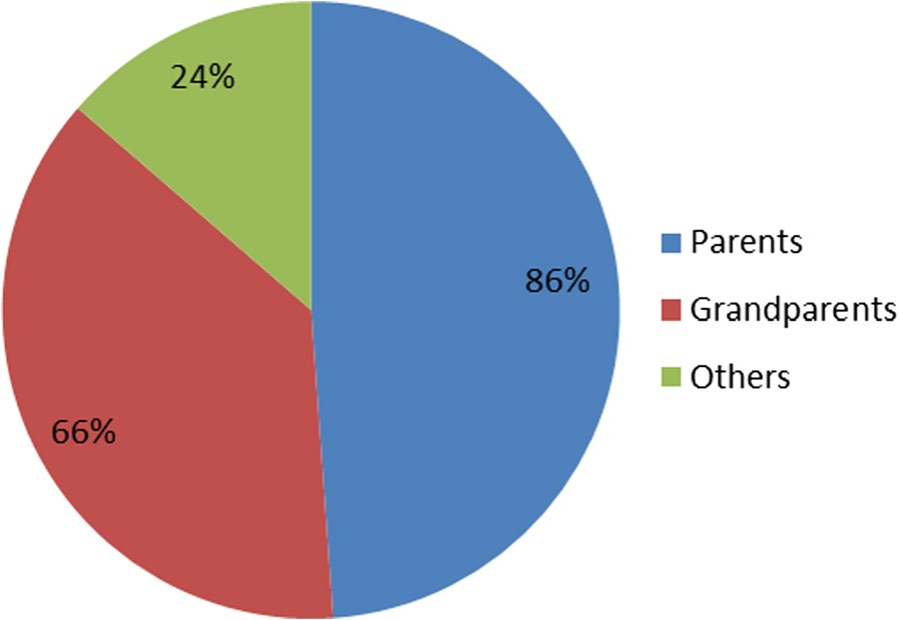
Sources of medicinal plant knowledge for management of TB remedy in Kitgum and Pader districts, Uganda

The most common method of preparation was by pounding and mixing concoction with water. The most common method of administration of the medicinal drug was oral intake. Cups were the most used unit for measuring dosage followed by teaspoon. Overall, 27% of respondents had knowledge about plants used to treat TB. Various sources of medicinal plant knowledge exist among the Acholi community. The majority (86%) of the respondents obtained plant knowledge from their parents (Fig. 4).

## Discussion

The present cross-sectional study demonstrates that indigenous knowledge and medicinal plants used in the management of TB still exist among the herbalists and non-herbalists in Kitgum and Pader districts despite the prolonged civil war that eroded the traditional culture of medicinal plants. Overall, only nine plant species were documented from a survey of 176 respondents. Although the number of species mentioned is small compared to what is recorded elsewhere in Uganda and other regions in Africa [10, 17, 32, 33], at least a database of the medicinal plants used for treating TB treatment in Kitgum and Pader district is initiated. However, the low number of plant species registered could imply that traditional knowledge for TB treatment is a closely guarded secret [34] and is likely a specialized practice, known only by few people. Knowledge of these plants may also be seen as a source of income to help lift the households out of poverty. Consequently, divulging this information would mean an infringement on their income source. Thus, to tap this knowledge, we suggest the introduction of alternative livelihoods opportunities for the target communities.

Some of the plant species documented in the present study have also been reported in other regions in Uganda and across the world for management of TB [10, 17, 35]. For example, *Zanthoxylum leprieuri* Guill. & Perr, *Steganotaenia araliaceae* Hochst and *Zanthoxylum chalybeum* Engl. have been documented as a remedy for managing symptoms of TB and other respiratory ailments in studies conducted in other parts of Uganda [10, 17]. This similarity in the usage of the same plant species from different places implies that these plants may be potential sources of anti-tuberculosis drugs. This calls for modern scientific validation to verify their efficacy and safety to promote their widespread use among the community.

The source of the medicinal plant knowledge from this study tally with what is reported in the literature [36], although parents were more resourceful than the grandparents. This shows that parents are still interested in passing indigenous knowledge to their children. This strengthens the model transfer of knowledge from grandparents to the parents who are now the main channel to the children according to our study. However, if adequate knowledge is not passed, to the parents then the indigenous knowledge can disappear as the older generation succumbs to death. The high ICF value (0.83) obtained indicates agreements among respondents on the different plant species used to manage TB [31]. Nonetheless, our results constitute potential plants for development of new drugs against *M. tuberculosis* which could be integrated into conventional medical therapies.

Rutaceae and Rubiaceae which were the dominant families in this study are in accordance with previous studies elsewhere in Uganda by Tabuti et al. [10] and Bunalema et al. [17] that have revealed these families as used in managing symptoms of TB and other respiratory infections. However, families Fabaceae and Asteraceae previously reported to house several medicinal plants for management of symptoms of TB by herbalists in other regions [10, 33] were not documented. This finding might be due to the high accessibility of these species in that region. To the best of our knowledge, plant species from the family Rubiaceae are mentioned for medicinal plant use for the first time in this region.

The finding of root bark as the most frequently mentioned plant part in managing symptoms of TB than other parts is consistent previous study conducted by Medikizela et al. [32] in O.R.Tambo district in the Eastern Cape Province, South Africa, in which roots (39%) were reported as the most frequently used plant part followed by bark (21%). On the contrary, other studies, e.g., Semenya and Maroyi, [37] in Limpopo province of South Africa; Bunalema et al. [17] in Mpigi and Bunamawa districts of Uganda and Lawal et al. [38] in Kenya have recorded leaves as the most frequently used plant part for preparing herbal remedies. The possible reasons for the preference of roots for herbal medicines are unknown but could be due to their high concentration of bioactive compounds and nutrients compared to other plant parts [39, 40]. However, the root collection is destructive and can pose serious threats to conservation of the plant species [41].

The most common form of utilization of herbal remedies was pounding and mixing concoction with cold or warm water. Similar findings have been reported in previous works conducted in other regions in East Africa [10, 17, 42] which indicated that decoction was the most prevalent mode of use of traditional ethnomedicine for TB management. The preference for decoction is probably because decoction is simple and results in more concentrations of phytochemicals and, therefore, might increase its bioactivity [43]. The use of a cup as the measure for dose administration reported in this study is in line with previous studies, e.g., Tuasha et al. [44] in Ethiopia. However, the use of cup as a measure of dosage is inappropriate and can cause overdose as cups are of different volumes. Therefore, there is a need to educate the herbalists to standardize their measurements.

## Conclusions

The study demonstrates the existence of medicinal plant knowledge and plants used in the community for managing symptoms of tuberculosis. However, for most of the plants mentioned the roots and the barks were the most used plant part for therapeutic purpose and this threatens the conservation of these plant species. Decoction was the common method of preparation. However, despite the small numbers of plants mentioned, the results of the current study could form a basis for the development of a new class of drugs for treatment of TB. There is need for a systematic and comprehensive inventory of these plants in the entire Acholi subregion to tap more information on these plants. Further isolation and purification of bioactive compounds responsible for the antimycobacterial activity, safety and efficacy of the mentioned plant species are recommended. The study also recommends any possible conservation method such as the establishment of medicinal plant home gardens and in vitro plant tissue culture.

## Limitations

An important limitation of this study is the small number of reported medicinal plants and this could imply that the respondents were not free to declare their acquired indigenous knowledge to the researchers for various reasons. In addition, the size of the cups which the healers referred to as used in the administration and the timing for administering the drugs in relation to the frequency were not fully captured.

## Data Availability

The data sets used and/or analysed during the current study are available from the corresponding author on reasonable request.

## Abbreviations

TB: Tuberculosis
HIV/AIDS: Human Immunodeficiency Virus/Acquired Immunodeficiency Syndrome
MDR-TB: Multi-drug resistant Tuberculosis
ICF: Informant Consensus Factor
UN: United Nations
UPDF: Uganda People’s Defense Forces
LRA: Lord’s Resistance Army
IDP: Internally Displaced People’s Camp
WHO: World Health Organization
APG: Angiosperm Phylogeny Group
